# Pathway Association Studies Reveal Gene Loci and Pathway Networks that Associated With Plasma Cystatin C Levels

**DOI:** 10.3389/fgene.2021.711155

**Published:** 2021-11-25

**Authors:** Hongxiao Jiao, Miaomiao Zhang, Yuan Zhang, Yaogang Wang, Wei-Dong Li

**Affiliations:** ^1^ Research Center of Basic Medical Sciences, Tianjin Medical University, Tianjin, China; ^2^ Department of Genetics, College of Basic Medical Sciences, Tianjin Medical University, Tianjin, China; ^3^ College of Public Health, Tianjin Medical University, Tianjin, China

**Keywords:** genome wide association, pathway association studies, genetics, United Kingdom biobank, cystatin C

## Abstract

As a marker for glomerular filtration, plasma cystatin C level is used to evaluate kidney function. To decipher genetic factors that control the plasma cystatin C level, we performed genome-wide association and pathway association studies using United Kingdom Biobank data. One hundred fifteen loci yielded *p* values less than 1 × 10^−100^, three genes (clusters) showed the most significant associations, including the *CST8-CST9* cluster on chromosome 20, the *SH2B3-ATXN2* gene region on chromosome 12, and the *SHROOM3*-*CCDC158* gene region on chromosome 4. In pathway association studies, forty significant pathways had FDR (false discovery rate) and or FWER (family-wise error rate) ≤ 0.001: spermatogenesis, leukocyte *trans*-endothelial migration, cell adhesion, glycoprotein, membrane lipid, steroid metabolic process, and insulin signaling pathways were among the most significant pathways that associated with the plasma cystatin C levels. We also performed Genome-wide association studies for eGFR, top associated genes were largely overlapped with those for cystatin C.

## Introduction

Chronic kidney disease (CKD) is a global public health problem with high morbidity (Coresh et al., 2003; Eckardt et al., 2013), which prevalence increases disproportionately with age (Coresh et al., 2007; Zhang et al., 2008; Stevens et al., 2010; Arora et al., 2013), and requires kidney dialysis or kidney transplantation to maintain life in the late stage (Levey et al., 2007; Jha et al., 2013). In addition to lifestyle and environmental factors, genetic factors play an important role in the progression of kidney disease (Kottgen et al., 2009). Genetic susceptibility exposures shared within families are associated with an increased risk of end-stage renal disease (ESRD) (Lei et al., 1998). Although mutation analysis has a high predictive value for monogenetic kidney diseases, finding genetic susceptibility variants for CKD by linkage mapping or candidate gene approaches has proven to be difficult (Hildebrandt, 2010).

Plasma cystatin C (encoded by *CST3* on 20p11.21), as an endogenous biomarker of kidney function (Onopiuk et al., 2015) and healthy aging (Sarnak et al., 2008), can effectively evaluate glomerular filtration rate (GFR) (Allen et al., 2015; Koksal et al., 2019). Cystatin C was found to be the most important biological features influencing organismal biological age (Gialluisi et al., 2021). In addition, cystatin C can inhibit the activity of extracellular cathepsins and participate in cardiovascular diseases (CVD), such as stroke, heart failure, coronary heart disease, and other pathological processes (Angelidis et al., 2013). Heritability of cystatin C that estimated by the Swedish twin model was 0.55 (0.49–0.60) in men, 0.63 (0.59–0.66) in women, and 0.60 (0.56–0.63) in both sexes combined, while the heritability of cystatin C estimated by genome-wide complex trait analysis (GCTA) was 0.40 in both sexes (Arpegard et al., 2015). Previous research on Genome-wide association studies (GWAS) have identified common genetic variants at the *CST3*, *UMOD*, *ATXN2,* and *STC1* loci that associated with cystatin C and its clinical derivatives (estimated glomerular filtration rate based on cystatin C) in European-ancestry subjects (Hwang et al., 2007; Kottgen et al., 2009; Chambers et al., 2010; Kottgen et al., 2010). SNPs in the *CST3* gene locus were associated with circulation concentration of cystatin C, while rs911119 showed the strongest association (Hwang et al., 2007; Kottgen et al., 2009; Chambers et al., 2010; Kottgen et al., 2010; van der Laan et al., 2016; Gorski et al., 2017). However, the minor allele of rs911119 merely explains 2.8% of the observed phenotypic variation in cystatin C (van der Laan et al., 2016). This may be because most of the common variants separately or corporately affect the traits/complex diseases with the modest effect (Hindorff et al., 2009), but higher penetrance rare variants were not detected by GWA studies. Minor genes that associated with complex traits usually become insignificant after adjustment for multiple testing. Meanwhile, interactions among genes were largely ignored, although non-additive effects (epistasis) may account for significant amount of heritabilities (Dong et al., 2003). As a result, most gene loci screened by GWAS cannot sufficiently explain the genetic variation of human traits or complex diseases.

To decipher genetic factors that associated with the plasma cystatin C level, and corporately consider multiple variations of gene-gene interactions in cystatin C, the modified GSEA method (GenGen Program) (Wang et al., 2007) was applied in our study. Genome-wide association and pathway association analyses were performed in United Kingdom Biobank subjects to find novel susceptibility loci and gene sets/pathways that associated with cystatin C levels. It was the largest GWAS for cystatin C in sample size, and the first genome wide pathway association study that ever carried out for cystatin C related phenotypes, providing more possibilities for further explaining the phenotypic variation of cystatin C.

## Materials and Methods

### Subjects

Subjects were originally collected to United Kingdom Biobank, which is an open access prospective cohort study in the United Kingdom. In 2006–2010, a total of 502,665 subjects from across the United Kingdom with age between 40 and 69 were recruited to participate in United Kingdom Biobank (Chua et al., 2019). Of these, we had cystatin C data for 465,309 individuals, outliers (>3 SD) were excluded thereafter. All participants gave written informed consent prior the study. The research protocol was authorized by the Human Ethics Committee of Tianjin Medical University. This research has been conducted using the United Kingdom biobank Resource under the project number of 45,676.

### Genotyping

Genomic DNA of participants was extracted from stored blood samples collected at United Kingdom Biobank. Genotyping was carried out by the United Kingdom Biobank Axiom^®^ Array (825,927 SNPs) or the Affymetrix United Kingdom BiLEVE Axiom^®^ Array (807,411 SNPs) and the methods have been described in detail (Welsh et al., 2017; Bycroft et al., 2018). In summary, samples call rates for inclusion was >80% and genotyped call rates was >95%. Quality control (QC) of the data was performed centrally at the Wellcome Trust Centre for Human Genetics (WTCHG) and genotype data are available for access to approved researchers, providing a powerful resource to decipher new genetic associations and the genetic bases of traits or complex diseases.

### Genome-wide Association Study and Gene-Based Association Study

Genome-wide quantitative association analyses were performed by PLINK 1.07 (Purcell et al., 2007) for cystatin C. SNPs with minor allele frequencies (MAF) < 0.05 and HWE *p* < 0.001were excluded, and Wald test of phenotype on allele dosage was performed for cystatin C association studies. GWAS were carried out in all subjects and in Caucasians, analyses for age and gender adjusted cystatin C were also performed (Table 1, Supplemental Table S2). *p* < 5 × 10^−8^ was regarded as significant to analyze interactions. To remove multiple SNPs which in linkage disequilibrium (LD), SNPs were also preselected with an LD-pruning method and those SNPs were deleted in the same LD block (*r* ^2^ > 0.6). IBD checking and adjustment of MDS component were performed in 5,000 randomly chosen Caucasian subjects. We also performed GWASs for eGFR, creatinine, and urea, respectively. Besides GWAS, we also conducted gene-based association study for cystatin C that implemented in MAGMA (de Leeuw et al., 2015), which analyzes multiple genetic variants to determine their joint effect and generates novel genome-wide signals at the gene-based level.

**TABLE 1:** Genome-wide association study for plasma cystatin C (*P* < 1 × 10^−150^).

CHR	SNP	Position (bp)	MAF	A1	A2	R2 (all)	T (all)	*p* (all)	*p* (Caucasian)	*p* (adjusted-Caucasian)^b^	SNP annotation	Gene (in or near)^c^	Distance^d^ (kb)
20	rs1158167	23578189	0.232	G	A	0.0369	−132.7	0^a^	0^a^	0^a^	intergenic	*CST9*	4.8
20	rs2983640	23586360	0.408	G	A	0.0155	−84.96	0^a^	0^a^	0^a^	missense mutation	*CST9*	
20	rs2983641	23586977	0.178	T	C	0.0024	33.15	1.29E-240	0^a^	0^a^	upstream	*CST9*	2.0
20	rs77114334	23565975	0.096	T	C	0.0022	31.6	6.35E-219	1.328E-188	1.201E-238	intergenic	*CST9*	17.0
20	rs2273378	23476389	0.115	G	A	0.0048	−46.87	0^a^	0^a^	0^a^	intronic	*CST8*	
20	rs3004118	23487503	0.462	T	C	0.0029	36.47	8.99E-291	6.102E-238	3.119E-288	non-coding variant	*CST8*	
20	rs73610708	23539846	0.085	T	C	0.0049	−46.87	0^a^	0^a^	0^a^	intergenic	*CST9L*	5.5
20	rs73102389	23635691	0.178	A	C	0.0043	44.5	0^a^	0^a^	0^a^	intergenic	*CST3*	21.3
20	rs112308292	23667865	0.122	A	C	0.0149	−83.18	0^a^	0^a^	0^a^	intronic	*CST4*	
20	rs13039144	23633755	0.177	G	A	0.0312	−121.6	0^a^	0^a^	0^a^	intergenic	*CST3*	19.4
20	rs66590796	23691153	0.111	T	G	0.0061	52.79	0^a^	0^a^	0^a^	intergenic	*CST4*	24.8
20	rs7266357	23700824	0.164	G	T	0.0029	36.51	2.42E-291	0^a^	0^a^	intergenic	*CST1*	27.3
20	rs6114264	23746310	0.137	T	C	0.0092	−64.74	0^a^	0^a^	0^a^	intergenic	*CSTP2*	7.1
20	rs67418849	23740511	0.091	A	G	0.0037	41.41	0^a^	0^a^	0^a^	intergenic	*CST1*	12.3
20	rs78916169	23405904	0.054	T	G	0.0021	30.97	1.99E-210	3.064E-187	2.144E-230	intergenic	*CSTL1*	14.4
20	rs761725	23402735	0.110	A	G	0.0017	28.06	4.35E-173	7.723E-144	1.093E-178	upstream	*NAPB*	2.0
20	rs2983608	23642839	0.205	C	T	0.0142	−81.47	0^a^	0^a^	0^a^	intronic	*LOC107985383*	
20	rs13040731	23642389	0.121	C	T	0.0018	−28.57	2.32E-179	9.755E-204	3.049E-242	intronic	*LOC107985383*	
20	rs6106728	23801950	0.210	T	C	0.0030	−37.41	8.9E-306	3.5E-305	0^a^	downstream	*LOC105372575*	0.5
20	rs3004096	23305768	0.334	T	G	0.0023	31.6	5.69E-219	3.233E-177	6.695E-209	intronic	*NXT1-AS1*	
20	rs6048704	23296209	0.251	T	C	0.0021	−31.18	3.44E-213	2.037E-207	5.996E-249	intronic	*NXT1-AS1*	
20	rs6049135	23783368	0.260	C	T	0.0020	30.17	9.34E-200	2.396E-154	2.918E-184	upstream	*LOC107985432*	2.0
12	rs653178	112007756	0.464	C	T	0.0024	33.05	3.49E-239	3.497E-169	5.568E-192	intronic	*ATXN2*	
12	rs3184504	111884608	0.464	T	C	0.0024	33.32	3.9E-243	4.023E-173	2.466E-195	missense mutation	*SH2B3*	
4	rs17319721	77368847	0.426	A	G	0.0024	33.37	8.61E-244	1.624E-203	7.16E-232	intronic	*SHROOM3*	
4	rs13106227	77418681	0.364	G	A	0.0016	−26.91	2.1E-159	3.242E-117	5.32E-124	intronic	*SHROOM3*	
4	rs907446	77254804	0.424	T	C	0.0017	27.63	6.96E-168	4.319E-113	9.297E-129	intronic	*CCDC158*	4.8

Associations with *p*-value < 1 × 10^−150^ are shown above. The chromosome (CHR) and base pair position are given with regards to the GRCh37 genome reference sequence. SNP, single nucleotide polymorphism; MAF, minor allele frequency; A1, Allele 1 code (minor allele); A2, Allele 2 code (major allele); R2, regression r-squared; T, Wald test (based on t-distribution). ^a^ *P*-value<1 × 10^−308^. ^b^ *p*-value for Caucasian adjusted for gender and age. ^c^Genes within 40 kb were based on RefSeq genes. ^d^ Distance from nearest genes to which they were annotated.

### Pathway Association Study

Pathway-based association studies were carried out by the GenGen program (Wang et al., 2007) based on the modified Gene Set Enrichment Algorithm (GSEA) (Lai et al., 2014) to further study the gene-gene interactions. The calculation process has been described in detail previously (Jiao et al., 2015). Briefly, for each gene, the SNP with the highest test statistic (chi-square detection/F-test) among all SNPs mapped to the gene was selected to represent the gene and all genes were ranked by sorting their statistic values from the largest to smallest. For any given gene set S, an enrichment score (ES) was calculated which reflects the overrepresentation of the gene set S at the forefront of the entire gene list. Then, in order to adjust the size of different genes, phenotype label permutation with 1,000 permutations was performed, and for each permutation, all the above calculations were repeated and the normalized enrichment score (NES) was calculated. The percentage of times that the ES value after permutation was greater than the actual ES value was called as “the empirical *p*-value”. Finally, a false-discovery rate (FDR) procedure was conducted to control the fraction of expected false-positive findings and family-wise error rate (FWER) ensured that there were no false positive gene sets in the output list. SNPs with minor allele frequencies (MAF) < 0.05 or Hardy-Weinberg equilibrium (HWE) < 0.001 were excluded and SNPs mapped at 20bp upstream or downstream of the gene region are regarded as part of the gene. A total of 377,863 SNPs that covered 21,787 genes were selected for Pathway-based association analyses. Meanwhile, 1,347 pathways/gene sets with size between 5 and 200 genes were selected from Kyoto Encyclopedia of Genes and Genomes (KEGG), BioCarta, and Gene Ontology (GO) databases. Empirical *p* values ≤0.001, the false-discovery rate (FDR) and or family-wise error rate (FWER) ≤ 0.001 were considered as significant.

## Results

We collected phenotype data of plasma cystatin C from subjects in the United Kingdom Biobank, 460,858 participants were included in our study after excluding outliers (>3 SD). The average level of cystatin C was 0.899 ± 0.137 mg/L (range, 0.386–1.436 mg/L). Distributions of cystatin C were shown in Supplemental Table S1 and Q-Q plots for distributions of cystatin C were shown in Supplemental Figure S2.

### Genome-wide Association Studies

**FIGURE 1:**
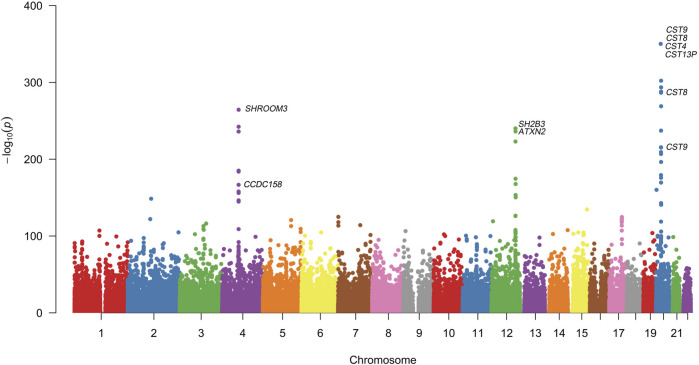
Manhattan Plot for genome wide association study of cystatin C. Variants are positioned according to the GRCh37 genome reference sequence. Associations with *p*-value < 1 × 10^−308^ were not displayed in normal scale.

**FIGURE 2:**
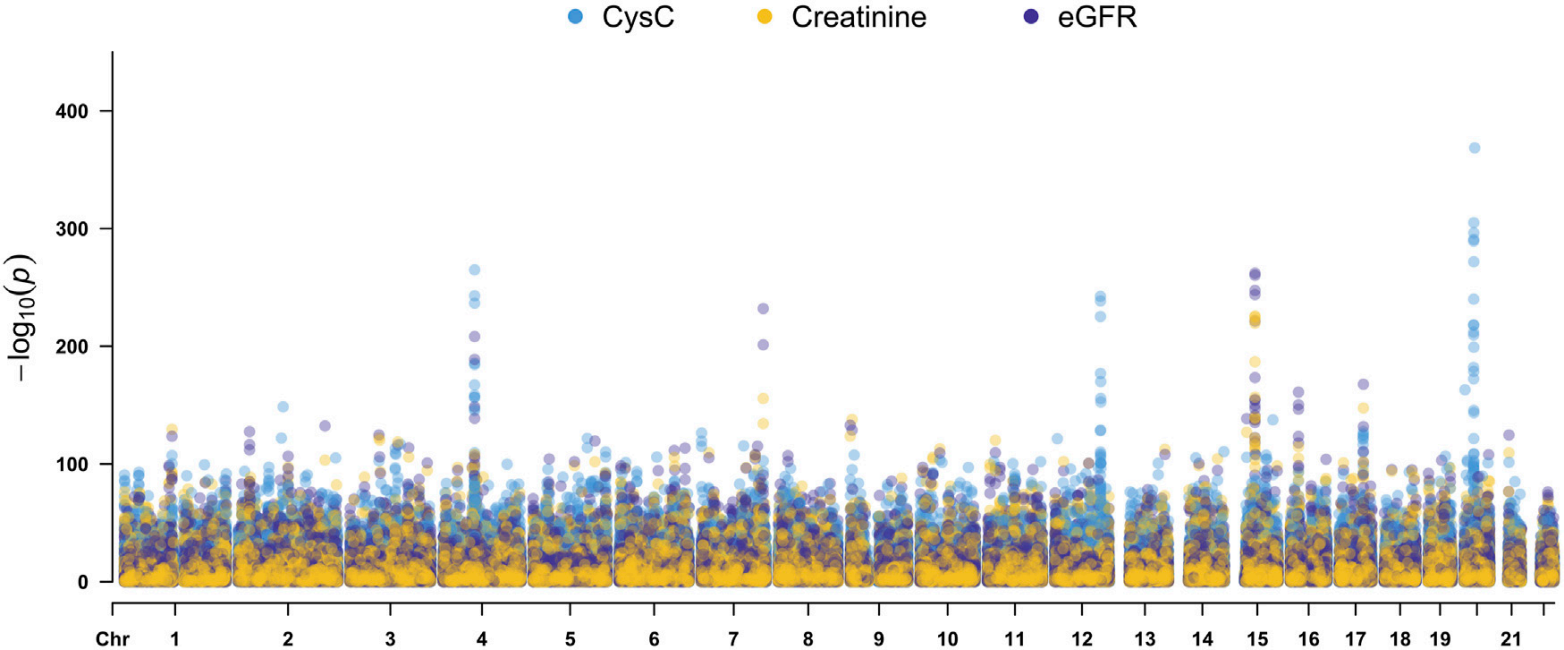
Manhattan Plots for cystatin C, creatinine, and eGFR genome wide association studies. Variants are positioned according to the GRCh37 genome reference sequence.

Quantitative GWAS analyses were performed for cystatin C in United Kingdom Biobank data. One hundred fifteen SNPs had *p* values less than 1 × 10^−100^, including significant associations in three gene regions: the *CST* gene cluster on 20p11.21, the *SHROOM3* -*CDCC158* region on 4q21.1, and the *SH2B3-ATXN2* region on 12q24.11. The gene regions are shown in the Manhattan plots (Figure 1). Analyses in Caucasians, as well as age and gender adjusted phenotypes, yielded similar results (Table 1, Supplemental Table S2). Many SNPs which significantly related to cystatin C overlapped with other renal functional phenotypes, including eGFR and creatinine (Figure 2). The most significant SNP for cystatin C levels was on rs1158167(*p*<1 × 10^−308^, Table 1 and Supplemental Table S2), near the cystatin C precursor gene family (*CST*3, *CST*4, *CST*9). However, no association was found for other renal function related phenotypes (creatinine, eGFR, urea) for *CST* cluster SNPs.

Two SNPs (rs17319721, and rs13106227) in the *SHROOM3* gene, one of the most replicated loci for renal function, reached a *p* < 1 × 100^−200^ (Table 1). The SNP rs17319721 yielded *p* = 8.61 × 10^−244^, *p* = 5.14 × 10^−209^, *p* = 5.65 × 10^−109^, and *p* = 1.51 × 10^−126^ for cystatin C, eGFR, creatinine, and urea, respectively (Supplemental Table S4). rs907446 in the *CCDC158* gene, which located in the upstream region of *SHROOM3*, was also associated with cystatin C (Table 1). A SNP rs653178 that located in the *SH2B3-ATXN2* gene region showed significant association (*p* = 3.49 × 10^−239^) with cystatin C (Table 1).

### Pathway Association Studies

**FIGURE 3:**
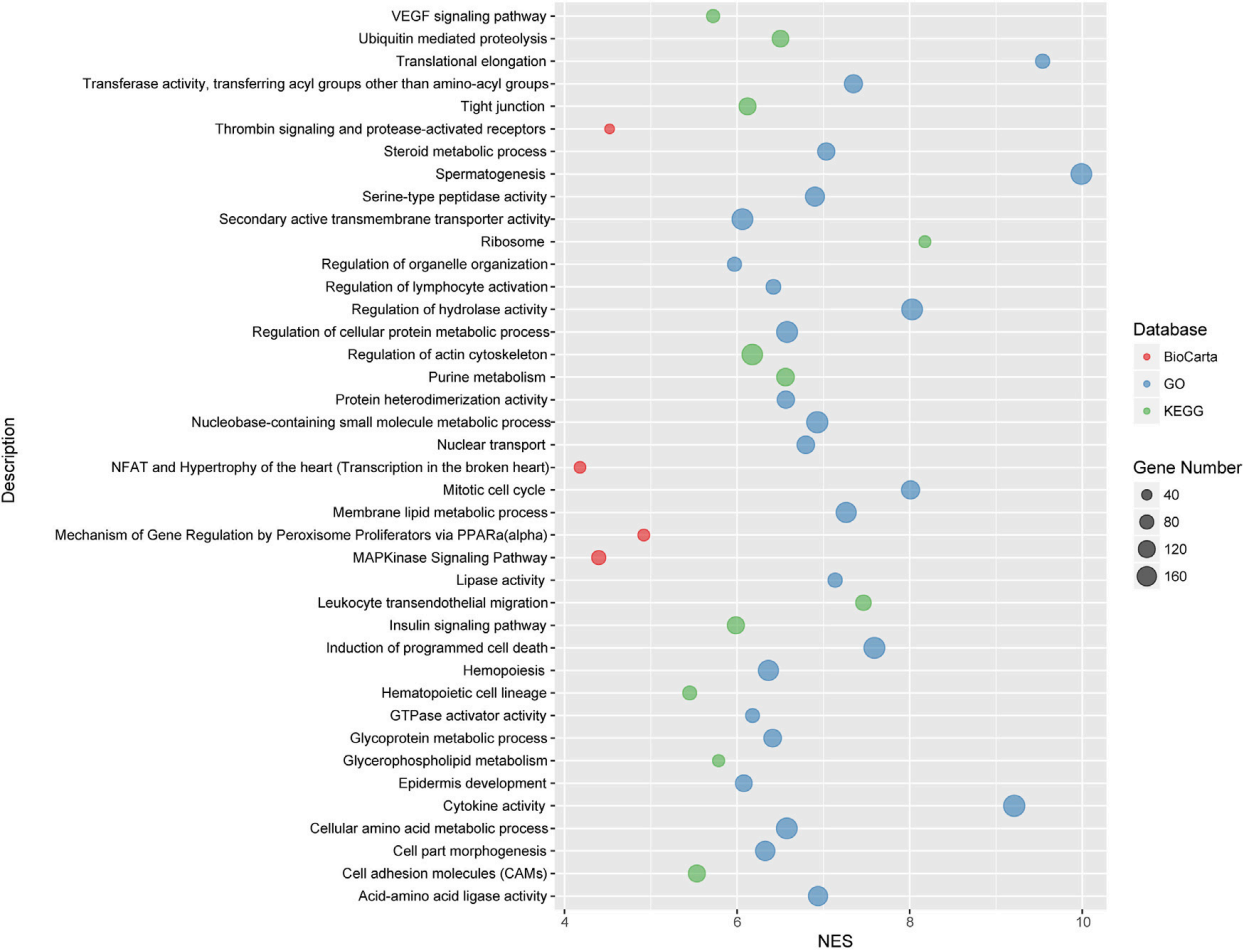
Top significant pathways/gene sets that associated with cystatin C levels. Results passing multiple hypothesis tests (false-discovery rate (FDR) and or family-wise error rate (FWER) ≤ 0.001) that identified by pathway association studies were shown in the figure. The NES(Wang et al., 2007) denotes normalized enrichment score (ES) which reflects the overrepresentation of gene sets at the top of the entire ranking list of genes in the genome.

We carried out pathway associations based on the GSEA algorithm. A total of 1,347 Kyoto Encyclopedia of Genes and Genomes (KEGG), BioCarta, and Gene Ontology (GO) pathways were tested. Figure 3 showed top significant pathways/gene sets that significantly associated with cystatin C levels. Pathways with false-discovery rate (FDR) and or family-wise error rate (FWER) ≤ 0.001 were set as significant (Table 2). Spermatogenesis, leukocyte transendothelial migration, cytoskeleton, tight junction, cell adhesion, glycoprotein, membrane lipid, steroid metabolic process, and insulin signaling pathways were among the most significant pathways.

**TABLE 2:** Pathway association studies for plasma cystatin C.

Pathway ID	Size	ES	NES	Empirical *P*	FDR	FWER	Database	Description
hsa03010	56	0.253	8.176	0.000	0.000	0.000	KEGG	Ribosome
hsa04670	99	0.424	7.464	0.000	0.000	0.000	KEGG	Leukocyte transendothelial migration
hsa00230	132	0.331	6.561	0.000	0.000	0.000	KEGG	Purine metabolism
hsa04120	115	0.304	6.503	0.000	0.000	0.000	KEGG	Ubiquitin mediated proteolysis
hsa04810	182	0.334	6.176	0.000	0.000	0.001	KEGG	Regulation of actin cytoskeleton
hsa04530	120	0.386	6.121	0.000	0.000	0.001	KEGG	Tight junction
hsa04910	124	0.305	5.986	0.000	0.000	0.001	KEGG	Insulin signaling pathway
hsa00564	58	0.410	5.786	0.000	0.000	0.001	KEGG	Glycerophospholipid metabolism
hsa04370	69	0.347	5.722	0.000	0.000	0.001	KEGG	VEGF signaling pathway
hsa04514	121	0.437	5.534	0.000	0.000	0.001	KEGG	Cell adhesion molecules (CAMs)
hsa04640	79	0.361	5.451	0.000	0.000	0.001	KEGG	Hematopoietic cell lineage
ppara Pathway	55	0.405	4.919	0.000	0.000	0.000	BioCarta	Mechanism of Gene Regulation by Peroxisome Proliferators via PPARa (alpha)
par1Pathway	35	0.514	4.523	0.001	0.001	0.002	BioCarta	Thrombin signaling and protease-activated receptors
mapk Pathway	81	0.313	4.397	0.000	0.001	0.003	BioCarta	Mapkinase Signaling Pathway
nfat Pathway	53	0.360	4.180	0.000	0.001	0.003	BioCarta	NFAT and Hypertrophy of the heart (Transcription in the broken heart)
GO0007283	185	0.315	9.989	0.000	0.000	0.000	GO	Spermatogenesis
GO0006414	82	0.256	9.540	0.000	0.000	0.000	GO	Translational elongation
GO0005125	195	0.315	9.211	0.000	0.000	0.000	GO	Cytokine activity
GO0051336	186	0.313	8.028	0.000	0.000	0.000	GO	Regulation of hydrolase activity
GO0007067	141	0.319	8.010	0.000	0.000	0.000	GO	Mitotic cell cycle
GO0012502	189	0.308	7.591	0.000	0.000	0.000	GO	Induction of programmed cell death
GO0016747	139	0.317	7.348	0.000	0.000	0.000	GO	Transferase activity, transferring acyl groups other than amino-acyl groups
GO0006643	174	0.300	7.264	0.000	0.000	0.000	GO	Membrane lipid metabolic process
GO0016298	83	0.406	7.136	0.000	0.000	0.000	GO	Lipase activity
GO0008202	126	0.340	7.033	0.000	0.000	0.000	GO	Steroid metabolic process
GO0016881	158	0.288	6.938	0.000	0.000	0.000	GO	Acid-amino acid ligase activity
GO0055086	194	0.285	6.929	0.000	0.000	0.000	GO	Nucleobase-containing small molecule metabolic process
GO0008236	155	0.300	6.902	0.000	0.000	0.000	GO	Serine-type peptidase activity
GO0051169	131	0.303	6.796	0.000	0.000	0.000	GO	Nuclear transport
GO0032268	189	0.296	6.579	0.000	0.000	0.000	GO	Regulation of cellular protein metabolic process
GO0006520	187	0.272	6.576	0.000	0.000	0.000	GO	Cellular amino acid metabolic process
GO0046982	127	0.357	6.565	0.000	0.000	0.000	GO	Protein heterodimerization activity
GO0051249	88	0.363	6.421	0.000	0.000	0.000	GO	Regulation of lymphocyte activation
GO0009100	133	0.372	6.412	0.000	0.000	0.000	GO	Glycoprotein metabolic process
GO0030097	175	0.344	6.363	0.000	0.000	0.000	GO	Hemopoiesis
GO0032990	161	0.377	6.326	0.000	0.000	0.000	GO	Cell part morphogenesis
GO0005099	78	0.430	6.179	0.000	0.000	0.000	GO	GTPase activator activity
GO0008544	117	0.333	6.078	0.000	0.000	0.000	GO	Epidermis development
GO0015291	187	0.339	6.062	0.000	0.000	0.000	GO	Secondary active transmembrane transporter activity
GO0033043	80	0.365	5.970	0.000	0.000	0.000	GO	Regulation of organelle organization

In pathway association studies, top forty (40) significant pathways/gene sets that significantly related to cystatin C levels were enriched from KEGG, GO, and BioCarta. ES, enrichment score; NES, normalized enrichment score; FDR, false-discovery rate; FWER, family-wise error rate.

## Discussion

Plasma cystatin C, as a superior endogenous biomarker of kidney function and healthy aging, is reported to be produced at a near constant rate and not to be affected by age, gender, or weight (Angelidis et al., 2013). However, there are limited data on genetic factors, that may influence plasma cystatin C levels.

Up to now, a few of GWAS studies on cystatin C and its clinical derivatives (estimated glomerular filtration rate based on cystatin C) have been reported (Hwang et al., 2007; Kottgen et al., 2009; Chambers et al., 2010; Kottgen et al., 2010; Gorski et al., 2017). From these reports, associations on *CST3*, *UMOD*, *ATXN2,* and *STC1* were identified. Of note, *CST3*, located in cystatin (CST) superfamily gene cluster on chromosome 20 and encoding the most abundant extracellular inhibitor of cysteine proteases, has associated with plasma cystatin C levels. SNPs that significantly related to cystatin C were identified, including rs1158167 (Hwang et al., 2007), rs13038305 (Kottgen et al., 2009) and rs911119 (Kottgen et al., 2010) on the CST gene cluster. Not unexpectedly, rs1158167 (*p* < 1 × 10^−308^, Table 1), the most significant common variation in our study, almost exclusively associated with cystatin C, meanwhile no association was found in other renal function related phenotypes (creatinine, eGFR, urea).

The *SH2B3-ATXN2* region on chromosomal 12q24.11 is another locus that we found related to cystatin C, which had associated with a wide spectrum of complex diseases, such as hematopoietic disorders of red and white blood cells, rheumatoid arthritis, retinal microcirculation problems, chronic kidney disease, cardiovascular infarction, and neurodegenerative diseases in previous studies (Gudbjartsson et al., 2009; Ikram et al., 2010; Kottgen et al., 2010; Mehta, 2011; Auburger et al., 2014). *SH2B3*, originally characterized as *LNK*, encodes a member of the SH2B adaptor family of proteins and is expressed in hematopoietic precursor cells and endothelial cells. It negatively regulates cytokine signaling involved in immune and inflammatory signaling pathways (Velazquez et al., 2002) and inhibits the JAK2/ABL1 dependent leukocyte proliferation. *SH2B3* mutations were associated with susceptibility to celiac disease type 13 and insulin-dependent diabetes mellitus.

*ATXN2* belongs to a group of genes that associated with microsatellite-expansion diseases and encodes cytoplasmic protein which modulates mTOR signals, modifying ribosomal translation and mitochondrial function. *ATXN2* deletions can lead to insulin resistance and obesity in mice (Meierhofer et al., 2016). The SNP rs653178, an intronic SNP at the *ATXN2* locus, reached significant association (*p* = 3.49 × 10^−239^, Table 1) with cystatin C levels in our study. This SNP is in near complete linkage disequilibrium (LD) with a missense SNP rs3184504 (*p* = 3.90 × 10^−243^, Table 1) at the *SH2B3* locus (r^2^=0.99). Indeed, several independent GWASs reported that rs653178 displayed significant associations with renal function phenotypes (estimated glomerular filtration rate based on cystatin C, eGFRcys) and CKD in populations of European and African ancestry (Kottgen, 2010; Kottgen et al., 2010; Boger and Heid, 2011; Liu et al., 2011). SNPs in these genes probably influence plasma levels of cystatin C and therefore eGFRcys, but not necessary for the true GFR susceptibility. Although rs653178 in *ATXN2* has no known functional effect, rs3184504 in *SH2B3* results in a substitution of tryptophan by arginine (R262 W) which is in the pleckstrin homology (PH) domain of *LNK* and induces changes in the structure and hydrophilic properties. At the same time, the missense variant also introduces a new phosphorylation site in the PH domain which may influence signaling pathways mediated by SH2B3. High conservation in mammals of the R262 residue in *LNK* suggests that it may have irreplaceable function in biological activities (Takaki et al., 2002). This may explain why the non-synonymous SNP in *SH2B3* seems to be a powerful pleiotropic variant involved in multiple complex diseases or traits including cystatin C.

Shroom family member 3 (*SHROOM3*) also appeared to be very significant in our GAWS studies on cystatin C. It plays an important role in mammalian kidney development and human kidney disease including CKD and ESRD through estimated glomerular filtration rate based on creatinine (eGFRcrea) (Kottgen et al., 2009; Boger et al., 2011). SHROOM3, the actin-binding protein, regulates the microtubule cytoskeleton during epithelial morphogenesis (Lee et al., 2007) and participates in epithelial cell arrangement and remodeling (Nishimura and Takeichi, 2008), which maintains the integrity of the glomerular filtration barrier (Yeo et al., 2015). Remarkably, rs13106227 (*p* = 5.32 × 10^−124^) in *SHROOM3* gene showed a strong correlation with cystatin C levels, which has not been reported by association research. However, the intronic SNP rs17319721 located in a highly conserved region in *SHROOM3* on chromosome 4 proved to be related to glomerular filtration rate (Kottgen et al., 2009) and impacted *cis*-expression of *SHROOM3* and renal allograft fibrosis (Menon et al., 2015). It is worth noting that SNP rs17319721 in *SHROOM3* showed consistency association across renal traits (cystatin C, eGFR, creatinine, and urea) in our study. The consistency of renal traits suggests that it may be an important functional site that interacts with other gene sites to perform its function. Another significant cystatin C associated SNP in *SHROOM3* was rs9992101 (not shown in Table 1)*,* which is in high LD with rs17319721 (r^2^ = 0.75) and both closely associated with CKD (Chambers et al., 2010).

Coiled-coil domain containing 158 (*CCDC158*) gene SNPs were significantly associated with cystatin C levels. *CCDC158*, located on 4q21.1, contains a conserved coiled-coil domain, which are found in important proteins involved in crucial interactions such as transcription factors (leucine zippers), molecular motors (myosin, kinesin), receptors, and signaling molecules (Lupas and Gruber, 2005). Unfortunately, there is little research on *CCDC158* related to the kidney due to its restricted expression in testis, even though coiled coils may change their oligomer state and conformation in response to environment changes. We need to point out these *CCDC158* gene region SNPs in *CDCC158* gene located within 100 Kb upstream of *SHROOM3* gene*,* still fell into the *SHROOM3* enhancer region.

To decipher genetic factors that control the plasma cystatin C levels and corporately consider multiple variation of gene-gene interactions in cystatin C, we performed pathway association analyses based on the genome-wide genotyping data in the United Kingdom Biobank database. We found 40 pathways/gene sets were significantly associated with cystatin C levels (Figure 3). It is worth noting that most significant pathways were cell adhesion, cell migration, and cell polarity related pathways, such as regulation of actin cytoskeleton, cell adhesion molecules, tight junction, and the VEGF signaling pathway.

Many metabolism related pathways were identified in our study, including cellular amino acid, glycoprotein, membrane lipid, steroid metabolic process, and the insulin signaling pathway. Furthermore, a few of immunity and inflammation-related pathways were involved, including leukocyte transendothelial migration and MAPKinase signaling pathways.

Cell adhesion plays an important role in maintaining the filtration integrity of epithelial cells (Sachs and Sonnenberg, 2013). Podocytes, as a key component of glomerular filtration barrier, adhere tightly to the underlying glomerular basement membrane (GBM), which provides cell adhesion and acts as solid-phase agonists of epithelial cells migration (Yurchenco, 2011). Previous research has reported that cystatin C was related to the pathological traits of invasiveness and angiogenesis caused by increased adhesion and migration in age-related macular degeneration (Carlsson et al., 2020), suggesting a relationship between cell adhesion and cystatin C levels. Indeed, our study of pathway association analysis in United Kingdom Biobank database revealed the genetic basis for its influence on the level of cystatin C. Whether cell adhesion affects the level of cystatin C and thus glomerular filtration needs further research.

Insulin is the major hormone controlling glucose and lipid metabolism and acts on most organs and cell types including the kidney, besides classical insulin target tissues (liver, skeletal muscle, and white adipose tissue) (Artunc et al., 2016). Abundant insulin receptors are expressed on podocytes, endothelial cells, mesangial cells, renal tubular epitheliums (Fornoni, 2010). Insulin signaling displays important roles in podocyte viability and renal tubular cell function (Artunc et al., 2016). Insulin resistance (IR) appears in the early stages of CKD patients, even if their GFR is still within the normal range, and becomes almost universal in those with the end stage of renal failure (Spoto et al., 2016). Impaired kidney function is well known to be linked with insulin resistance, however, the causality of this relationship was not well defined. The insulin signaling cascades in kidney mainly includes phosphoinoside-3-kinase (PI3K), mitogen-activated protein kinase (MAPK), nuclear factor kappa B (NF-κB), glucose transporter 4 (GLUT4), and adenosine monophosphate activated protein kinase (AMPK). Clinically, a higher level of serum cystatin C was associated with decreased insulin sensitivity in type 1 diabetic patients (Uruska et al., 2014). Although whether the level of cystatin C can accurately reflect insulin resistance remains to be verified, the role of insulin signaling pathway which was found in pathway-based association analyses cannot be ignored as the genetic background of cystatin C.

In summary, recent technological advancements resulted in large collections of whole-genome genotyping data which provides better coverage and increased imputation quality. It was the largest GWAS for cystatin C in sample size, and the first genome wide pathway association study for cystatin C that ever carried out. We have also found novel associations and pathways for plasma cystatin C levels. However, under current conditions, we are unable to verify biological functions of results from our pathway research, further verifications in the transcriptome dataset are needed in the future.

## Conclusion

We performed genome wide association and pathway association studies on cystatin C in an open access prospective cohort study. We have identified three major gene loci and 40 novel biological pathways, including cell adhesion and insulin signaling pathways, that associated with the plasma cystatin C levels. Our results provided more genetic bases for further understanding the phenotypic variations of cystatin C.

## Data Availability

The datasets presented in this study can be found in online repositories. The names of the repository/repositories and accession number(s) can be found in the article/Supplementary Material.
