# Supplementary material for: Pathway Association Studies Reveal Gene Loci and Pathway Networks that Associated With Plasma Cystatin C Levels
Source: Front Genet. 2021 Nov 25;12:711155. doi: 10.3389/fgene.2021.711155 (PMC8656399; doi:10.3389/fgene.2021.711155)
Supplement: Supplementary file 1 [file DataSheet1.docx]

Supplementary Material

# Supplemental Figure S1: Q-Q plots for distributions of plasma cystatin C levels in 460 858 participants.

# Outliers (>3 SD) were excluded. The line shows the expected distribution under the null hypothesis of no association at any locus.


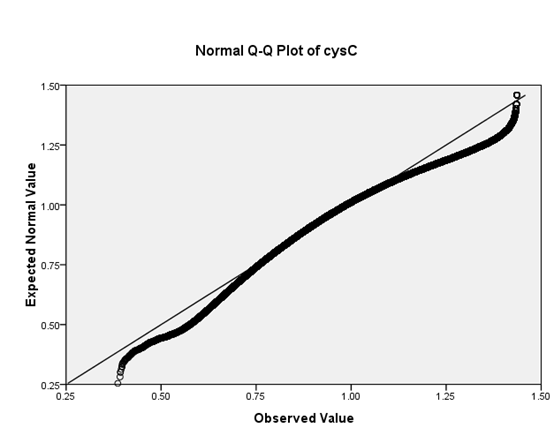


# Supplemental Table S1：Distribution of cystatin C levels in United Kingdom individuals.

|  | **N** | **Min**  **(mg/L)** | **Max**  **(mg/L)** | **Mean**  **(mg/L)** | **Std**^a^ | **Skewness** | **Kurtosis** |
| --- | --- | --- | --- | --- | --- | --- | --- |
| ALL | 460858 | 0.386 | 1.436 | 0.899 | 0.137 | 0.635 | 0.708 |
| Male | 211023 | 0.403 | 1.485 | 0.933 | 0.134 | 0.753 | 1.068 |
| Female | 249756 | 0.378 | 1.377 | 0.870 | 0.134 | 0.647 | 0.599 |

^a^Outliers (>3 SD) were respectively excluded in all, male and female in our study. N, number of individuals; Min, minimum; Max, maximum; Std, standard deviation.

**Supplemental Table S2：****Genome-wide association studies** **for** **plasma cystatin C.**

| **CHR** | **SNP** | **Position**  **(bp)** | **MAF** | **A1** | **A2** | **R2**  **(all)** | **T**  **(all)** | ***P***  **(all)** | ***P***  **(Caucasian)** | ***P* (adjusted-**  **Caucasian) ^b^** | **SNP**  **annotation** | **Gene**  **(in or near) ^c^** | **distance ^d^** |
| --- | --- | --- | --- | --- | --- | --- | --- | --- | --- | --- | --- | --- | --- |
| 20 | rs1158167 | 23578189 | 0.232 | G | A | 0.0369 | -132.7 | 0^a^ | 0^a^ | 0^a^ | intergenic | *CST9* | 4.8kb |
| 20 | rs13039144 | 23633755 | 0.177 | G | A | 0.0312 | -121.6 | 0^a^ | 0^a^ | 0^a^ | intergenic | *CST3* | 19.4kb |
| 20 | rs6114264 | 23746310 | 0.137 | T | C | 0.0092 | -64.74 | 0^a^ | 0^a^ | 0^a^ | intergenic | *CSTP2* | 7.1kb |
| 20 | rs66590796 | 23691153 | 0.111 | T | G | 0.0061 | 52.79 | 0^a^ | 0^a^ | 0^a^ | intergenic | *CST4* | 24.8kb |
| 20 | rs67418849 | 23740511 | 0.091 | A | G | 0.0037 | 41.41 | 0^a^ | 0^a^ | 0^a^ | intergenic | *CST1* | 12.3kb |
| 20 | rs73102389 | 23635691 | 0.178 | A | C | 0.0043 | 44.5 | 0^a^ | 0^a^ | 0^a^ | intergenic | *CST3* | 21.3kb |
| 20 | rs73610708 | 23539846 | 0.085 | T | C | 0.0049 | -46.87 | 0^a^ | 0^a^ | 0^a^ | intergenic | *CST9L* | 5.5kb |
| 20 | rs2983608 | 23642839 | 0.205 | C | T | 0.0142 | -81.47 | 0^a^ | 0^a^ | 0^a^ | intronic | *LOC107985383* |  |
| 20 | rs2983640 | 23586360 | 0.408 | G | A | 0.0155 | -84.96 | 0^a^ | 0^a^ | 0^a^ | missense mutation | *CST9* |  |
| 20 | rs2273378 | 23476389 | 0.115 | G | A | 0.0048 | -46.87 | 0^a^ | 0^a^ | 0^a^ | intronic | *CST8* |  |
| 20 | rs112308292 | 23667865 | 0.122 | A | C | 0.0149 | -83.18 | 0^a^ | 0^a^ | 0^a^ | intronic | *CST4* |  |
| 20 | rs6106728 | 23801950 | 0.21 | T | C | 0.003 | -37.41 | 8.9E-306 | 3.5E-305 | 0a | downstream | *LOC105372575* | 0.5kb |
| 20 | rs7266357 | 23700824 | 0.164 | G | T | 0.0029 | 36.51 | 2.42E-291 | 0^a^ | 0^a^ | intergenic | *CST1* | 27.3kb |
| 20 | rs3004118 | 23487503 | 0.462 | T | C | 0.0029 | 36.47 | 8.99E-291 | 6.102E-238 | 3.119E-288 | non-coding variant | *CST8* |  |
| 4 | rs17319721 | 77368847 | 0.426 | A | G | 0.0024 | 33.37 | 8.61E-244 | 1.624E-203 | 7.16E-232 | intronic | *SHROOM3* |  |
| 12 | rs3184504 | 111884608 | 0.464 | T | C | 0.0024 | 33.32 | 3.9E-243 | 4.023E-173 | 2.466E-195 | missense mutation | *SH2B3* |  |
| 20 | rs2983641 | 23586977 | 0.178 | T | C | 0.0024 | 33.15 | 1.29E-240 | 0^a^ | 0^a^ | upstream | *CST9* | 2kb |
| 12 | rs653178 | 112007756 | 0.464 | C | T | 0.0024 | 33.05 | 3.49E-239 | 3.497E-169 | 5.568E-192 | intronic | *ATXN2* |  |
| 20 | rs3004096 | 23305768 | 0.334 | T | G | 0.0023 | 31.6 | 5.69E-219 | 3.233E-177 | 6.695E-209 | intronic | *NXT1-AS1* |  |
| 20 | rs77114334 | 23565975 | 0.096 | T | C | 0.0022 | 31.6 | 6.35E-219 | 1.328E-188 | 1.201E-238 | intergenic | *CST9* | 17.0kb |
| 20 | rs6048704 | 23296209 | 0.251 | T | C | 0.0021 | -31.18 | 3.44E-213 | 2.037E-207 | 5.996E-249 | intronic | *NXT1-AS1* |  |
| 20 | rs78916169 | 23405904 | 0.054 | T | G | 0.0021 | 30.97 | 1.99E-210 | 3.064E-187 | 2.144E-230 | intergenic | *CSTL1* | 14.4kb |
| 20 | rs6049135 | 23783368 | 0.26 | C | T | 0.002 | 30.17 | 9.34E-200 | 2.396E-154 | 2.918E-184 | upstream | *LOC107985432* | 2.0kb |
| 20 | rs1541237 | 23258976 | 0.164 | T | C | 0.0018 | -28.86 | 4.97E-183 | 1.866E-57 | 5.095E-64 | unknown |  |  |
| 20 | rs13040731 | 23642389 | 0.121 | C | T | 0.0018 | -28.57 | 2.32E-179 | 9.755E-204 | 3.049E-242 | intronic | *LOC107985383* |  |
| 12 | rs11065987 | 112072424 | 0.4 | G | A | 0.0018 | 28.43 | 1.17E-177 | 7.213E-122 | 2.268E-138 | intergenic | *BRAP* | 7.5kb |
| 20 | rs761725 | 23402735 | 0.11 | A | G | 0.0017 | 28.06 | 4.35E-173 | 7.723E-144 | 1.093E-178 | upstream | *NAPB* | 2.0kb |
| 12 | rs17630235 | 112591686 | 0.392 | A | G | 0.0017 | 27.87 | 8.8E-171 | 1.507E-116 | 3.306E-133 | downstream | *TRAFD1* | 0.5kb |
| 4 | rs907446 | 77254804 | 0.424 | T | C | 0.0017 | 27.63 | 6.96E-168 | 4.319E-113 | 9.297E-129 | intronic | *CCDC158* |  |
| 20 | rs6132532 | 2315543 | 0.05 | G | A | 0.0016 | -27.3 | 5.27E-164 | 0.3535 | 0.8194 | intronic | *TGM3* |  |
| 4 | rs13106227 | 77418681 | 0.364 | G | A | 0.0016 | -26.91 | 2.1E-159 | 3.242E-117 | 5.32E-124 | intronic | *SHROOM3* |  |
| 12 | rs17696736 | 112486818 | 0.412 | G | A | 0.0015 | 26.65 | 2.23E-156 | 7.366E-102 | 3.728E-116 | intronic | *NAA25* |  |
| 12 | rs11066301 | 112871372 | 0.409 | G | A | 0.0015 | 26.39 | 2.15E-153 | 1.055E-99 | 4.334E-114 | intronic | *PTPN11* |  |
| 2 | rs260687 | 109578855 | 0.081 | C | T | 0.0016 | -26.04 | 2.48E-149 | 0.04237 | 0.06146 | intronic | *EDAR* |  |
| 20 | rs4627645 | 23740591 | 0.303 | C | T | 0.0014 | 25.76 | 2.91E-146 | 5.455E-151 | 8.005E-185 | intergenic | *CST1* | 12.4kb |
| 20 | rs6114382 | 23932584 | 0.104 | G | T | 0.0014 | -25.61 | 1.62E-144 | 1.889E-63 | 1.234E-71 | intergenic | *CSTP1* | 33.1kb |
| 15 | rs7170666 | 89154966 | 0.052 | A | G | 0.0014 | -25.05 | 2E-138 | 0.007719 | 0.02376 | intronic | *AEN* |  |
| 12 | rs4766897 | 112179471 | 0.323 | T | C | 0.0013 | 24.21 | 2.23E-129 | 5.1E-91 | 4.043E-101 | intronic | *ACAD10* |  |
| 12 | rs1265565 | 111715197 | 0.361 | T | C | 0.0013 | 24.18 | 4.04E-129 | 7.319E-87 | 1.178E-95 | intronic | *CUX2* |  |
| 17 | rs11868441 | 59239221 | 0.198 | A | G | 0.0013 | -24.08 | 4.7E-128 | 1.145E-33 | 2.402E-40 | intronic | *BCAS3* |  |
| 7 | rs12702509 | 1281064 | 0.261 | G | A | 0.0012 | -23.95 | 1.06E-126 | 1.373E-39 | 8.58E-45 | intergenic | *UNCX* | 8.4kb |
| 17 | rs8064787 | 58977757 | 0.156 | C | T | 0.0012 | -23.77 | 8.89E-125 | 2.027E-35 | 1.095E-39 | intronic | *BCAS3* |  |
| 2 | rs10171578 | 105266464 | 0.061 | C | A | 0.0012 | -23.57 | 8.74E-123 | 0.2627 | 0.9014 | unknown |  |  |
| 17 | rs9906672 | 58845031 | 0.095 | C | T | 0.0012 | -23.57 | 1.01E-122 | 7.256E-18 | 3.818E-20 | intronic | *BCAS3* |  |
| 5 | rs7717322 | 132074890 | 0.107 | A | G | 0.0012 | -23.54 | 2.04E-122 | 0.09833 | 0.1893 | upstream | *KIF3A* | 2.0kb |
| 20 | rs73102333 | 23623917 | 0.217 | A | G | 0.0012 | 23.53 | 2.65E-122 | 2.71E-101 | 4.306E-116 | intergenic | *CST3* | 9.6kb |
| 12 | rs11064432 | 6968741 | 0.058 | G | C | 0.0012 | -23.51 | 3.51E-122 | 0.9775 | 0.582 | intronic | *USP5* |  |

*P*-value<1×10^-120^ of SNPs significantly related to cystatin C levels are shown above. The chromosome (CHR) and base pair position are given with regards to the GRCh37 genome reference sequence. SNP, single nucleotide polymorphism; MAF, minor allele frequency; A1, Allele 1 code (minor allele); A2, Allele 2 code (major allele); R2, regression r-squared; T, Wald test (based on t-distribution). **^a^***P*-value<1×10^-308^. **^b^** *P*-value for Caucasian adjusted for gender and age. **^c^** Genes within 40kb were based on RefSeq genes. **^d^** Distance from nearest genes to which they were annotated.

**Supplemental Table S3：****Gene-based Association Study for plasma cystatin C.**

| **Gene Symbol** | **Gene ID** | **CHR** | **START** | **STOP** | **NSNPS** | **N** | ***P*-value** |
| --- | --- | --- | --- | --- | --- | --- | --- |
| *CST4* | 1472 | 20 | 23666277 | 23669662 | 1 | 432615 | 1E-50 |
| *CST8* | 10047 | 20 | 23471766 | 23476655 | 1 | 433536 | 1E-50 |
| *CST9* | 128822 | 20 | 23583047 | 23586610 | 1 | 433727 | 1E-50 |
| *UNCX* | 340260 | 7 | 1272654 | 1276613 | 1 | 410659 | 2.7E-37 |
| *CCDC158* | 339965 | 4 | 77234192 | 77333285 | 25 | 403268 | 1.5883E-10 |
| *ACAD10* | 80724 | 12 | 112123857 | 112194911 | 4 | 432689 | 5E-10 |
| *ATXN2* | 6311 | 12 | 111890018 | 112037480 | 4 | 433252 | 5E-10 |
| *BCAS3* | 54828 | 17 | 58755172 | 59470199 | 68 | 429937 | 5E-10 |
| *BRAP* | 8315 | 12 | 112079950 | 112123790 | 2 | 431500 | 5E-10 |
| *CST1* | 1469 | 20 | 23728190 | 23731574 | 2 | 433872 | 5E-10 |
| *CST9L* | 128821 | 20 | 23545369 | 23549386 | 2 | 433015 | 5E-10 |
| *CSTL1* | 128817 | 20 | 23420322 | 23425567 | 3 | 433826 | 5E-10 |
| *CUX2* | 23316 | 12 | 111471828 | 111788358 | 30 | 427270 | 5E-10 |
| *NAA25* | 80018 | 12 | 112464493 | 112546826 | 5 | 433276 | 5E-10 |
| *NAPB* | 63908 | 20 | 23355156 | 23402156 | 6 | 432866 | 5E-10 |
| *PTPN11* | 5781 | 12 | 112856536 | 112947717 | 5 | 424187 | 5E-10 |
| *SH2B3* | 10019 | 12 | 111843720 | 111889427 | 2 | 433759 | 5E-10 |
| *SHROOM3* | 57619 | 4 | 77356253 | 77704406 | 64 | 429691 | 5E-10 |

CHR, chromosome; START, start position of the gene; STOP, stop position of the gene; NSNPS, the number of SNPs annotated to that gene that were found in the data and were not excluded based on internal SNP QC; N, the sample size used when analyzing that gene.

**Supplemental Table S4：Top significant SNPs for eGFR, creatinine and urea by genome wide association study.**

| **TRAIT** | **CHR** | **SNP** | **Position(bp)** | **MAF** | **A1** | **A2** | **R2** | **T** | ***P*-value** | **SNP**  **annotation** | **Gene**  **(in or near) ^c^** | **distance ^d^** |
| --- | --- | --- | --- | --- | --- | --- | --- | --- | --- | --- | --- | --- |
| eGFR | 15 | rs1153849 | 45695695 | 0.274 | A | G | 0.003 | -34.66 | 7.237E-263 | missense mutation | *SPATA5L1* |  |
|  | 15 | rs1288775 | 45661678 | 0.273 | A | T | 0.003 | -34.54 | 4.687E-261 | missense mutation | *GATM* |  |
|  | 15 | rs1145086 | 45654327 | 0.390 | G | A | 0.002 | -33.67 | 3.269E-248 | missense mutation | *GATM* |  |
|  | 7 | rs10224002 | 151415041 | 0.286 | G | A | 0.002 | -32.60 | 8.44E-233 | intronic | *PRKAG2* |  |
|  | 7 | rs7805747 | 151407801 | 0.272 | A | G | 0.002 | -30.34 | 5.24E-202 | intronic | *PRKAG2* |  |
|  | 4 | rs17319721 | 77368847 | 0.426 | A | G | 0.002 | -30.87 | 5.135E-209 | intronic | *SHROOM3* |  |
|  | 4 | rs13146355 | 77412140 | 0.441 | A | G | 0.002 | -29.38 | 1.38E-189 | intronic | *SHROOM3* |  |
|  | 17 | rs7219624 | 59460164 | 0.077 | A | G | 0.002 | -27.68 | 1.772E-168 | intronic | *BCAS3* |  |
|  | 16 | rs12917707 | 20367690 | 0.176 | T | G | 0.002 | 27.12 | 8.096E-162 | upstream | *UMOD* | 2.0kb |
|  | 16 | rs111285796 | 20361087 | 0.180 | TTCTGCCAGA | T | 0.001 | 26.19 | 4.586E-151 | splice donor variant | *UMOD* |  |
| creatinine | 15 | rs1153849 | 45695695 | 0.274 | A | G | 0.002 | 32.14 | 1.996E-226 | missense mutation | *SPATA5L1* |  |
|  | 15 | rs1288775 | 45661678 | 0.273 | A | T | 0.002 | 32.10 | 7.963E-226 | missense mutation | *GATM* |  |
|  | 15 | rs1145086 | 45654327 | 0.390 | G | A | 0.002 | 31.92 | 2.83E-223 | missense mutation | *GATM* |  |
|  | 7 | rs10224002 | 151415041 | 0.286 | G | A | 0.002 | 26.65 | 2.145E-156 | intronic | *PRKAG2* |  |
|  | 7 | rs7805747 | 151407801 | 0.272 | A | G | 0.001 | 24.74 | 4.609E-135 | intronic | *PRKAG2* |  |
|  | 17 | rs7219624 | 59460164 | 0.077 | A | G | 0.001 | 25.95 | 2.572E-148 | intronic | *BCAS3* |  |
|  | 15 | rs1060896 | 45554267 | 0.348 | C | A | 0.001 | 25.26 | 1.242E-140 | missense mutation | *SLC28A2* |  |
|  | 15 | rs11854325 | 45548959 | 0.347 | T | C | 0.001 | 25.16 | 1.303E-139 | intronic | *SLC28A2* |  |
|  | 16 | rs12917707 | 20367690 | 0.176 | T | G | 0.001 | -22.86 | 1.5E-115 | upstream | *UMOD* | 2.0kb |
|  | 4 | rs17319721 | 77368847 | 0.426 | A | G | 0.001 | 22.18 | 5.647E-109 | intronic | *SHROOM3* |  |
| urea | 20 | rs6132532 | 2315543 | 0.051 | G | A | 0.003 | -38.63 | 0^a^ | intronic | *TGM3* |  |
|  | 5 | rs35397 | 33951116 | 0.093 | G | T | 0.003 | -39.90 | 0^a^ | intronic | *SLC45A2* |  |
|  | 5 | rs16891982 | 33951693 | 0.069 | C | G | 0.005 | -48.82 | 0^a^ | missense mutation | *SLC45A2* |  |
|  | 5 | rs28777 | 33958959 | 0.058 | C | A | 0.005 | -46.53 | 0^a^ | intronic | *SLC45A2* |  |
|  | 7 | rs6950388 | 1270699 | 0.222 | G | A | 0.002 | -32.83 | 3.92E-236 | upstream | *UNCX* | 2.0kb |
|  | 4 | rs13146355 | 77412140 | 0.441 | A | G | 0.001 | 25.36 | 9.113E-142 | intronic | *SHROOM3* |  |
|  | 4 | rs17319721 | 77368847 | 0.426 | A | G | 0.001 | 23.94 | 1.505E-126 | intronic | *SHROOM3* |  |
|  | 7 | rs7805747 | 151407801 | 0.272 | A | G | 0.001 | 23.11 | 3.834E-118 | intronic | *PRKAG2* |  |
|  | 7 | rs10224002 | 151415041 | 0.286 | G | A | 0.001 | 22.03 | 1.805E-107 | intronic | *PRKAG2* |  |
|  | 17 | rs7219624 | 59460164 | 0.077 | A | G | 0.001 | -22.40 | 4.358E-111 | intronic | *BCAS3* |  |

Creatinine and urea phenotype data were obtained from the UK Biobank; glomerular filtration rate (GFR) was estimated using the simplified Modification of Diet in Renal Disease Study equation. MAF, Minor Allele Frequency; A1, Allele 1 code (minor allele); A2, Allele 2 code (major allele); R2, regression r-squared; T, Wald test (based on t-distribution); *P*-value, Wald test asymptotic *p*-value. ^a^*P*-value<1×10^-263^. **^b^** Genes within 40kb were based on RefSeq genes. **^d^** Distance from nearest genes to which they were annotated.
